# Surgeon-performed ultrasound-guided head and neck procedures during COVID-19 pandemic

**DOI:** 10.1186/s43163-022-00258-2

**Published:** 2022-06-04

**Authors:** Liew Yew Toong, Sakina Ghauth

**Affiliations:** Otorhinolaryngology (ENT) Department, Faculty of Medicine, Universiti Malaya, Jalan Universiti, 59100 Kuala Lumpur, Malaysia

**Keywords:** Surgeon-performed ultrasound, Ultrasound-guided procedures, COVID-19 pandemic

## Abstract

**Background:**

The ultrasonographic procedure is a common diagnostic and therapeutic method in daily medical practice. Surgeon-performed ultrasound (SUS) has been gaining popularity, especially during this COVID-19 pandemic.

**Methods:**

Two ENT surgeons performed US-guided diagnostic or therapeutic procedures in the outpatient clinic. A successful SUS is defined as the diagnostic procedure's ability to facilitate the final histopathological diagnosis that can guide the subsequent treatment and disease resolution following the therapeutic procedure without the need for more invasive open surgical intervention.

**Results:**

Out of 10 participants, 6 are males, and 4 are females. There were no complications noted. All subjects had successful tissue sampling or intervention on the first attempt for diagnostic and therapeutic SUS.

**Conclusions:**

With COVID-19, there has been a significant shift in ordinary medical practice. SUS is proven to be a safe and effective method to facilitate the management of head and neck pathologies.

## Background

The advent of small, portable, high-resolution ultrasound machines has resulted in a paradigm shift in managing head and neck pathologies. Surgeons have the advantage of direct access to high-quality, surgery-specific, and treatment-directed imaging.

Flatman et al [1] The American Academy of Otolaryngology-Head and Neck Surgery endorses surgeon-performed ultrasound (SUS) and supports its use in the head and neck [2]. It allows immediate therapeutic interventions such as ultrasound-guided needle aspiration of abscesses, and in terms of diagnostic value, it is safe and decreases both times to definitive management and the number of patient visits [3, 4].

The coronavirus (COVID-19) pandemic caused by the novel severe acute respiratory syndrome coronavirus 2 (SARS-CoV-2) has affected the world since early 2020. On March 11th, 2020, the World Health Organization (WHO) declared COVID-19 a global pandemic, leading to widespread economic, social, and health turmoil [5]. As a result, this has severely impacted how medicine is practiced now. In Malaysia, the number of cases has been surging exponentially since early 2021 and has led to a total death toll exceeding 22,000 as of mid-September 2021 [6]. In addition, this virus’ high risk of contamination in healthcare teams has imparted fear to healthcare professionals and patients. Consequently, essential urgent or semi-urgent sonographic guided procedures conventionally done by radiologists need to be postponed. Ergo, this study objective is to report on the first introduction of SUS to a dedicated head and neck cancer clinic in Malaysia as a diagnostic and therapeutic tool in our center.

## Methods

### Patients

We conducted a retrospective, observational study of patients referred to the Otorhinolaryngology-Head and Neck Surgery Clinic at Universiti Malaya Medical Centre, Kuala Lumpur. They were divided into two different groups which are therapeutic and diagnostic. The therapeutic group is defined as a subject with diseases that requires a sonographic-guided injection as part of the treatment. On the other hand, the diagnostic group is for subjects who need the sonographic guided biopsy as part of their disease investigation. For both groups, we enrolled adult patients older than 18 years from June 2021 to September 2021; for the diagnostic group, the inclusion criteria include subjects who presented with suspicious head and neck masses of more than 2 cm or unresolved suspicious cervical neck nodes, while inclusion criteria for the therapeutic group are subject with diseases which requires a sonographic-guided injection as part of their treatment. These subjects were also selected when there was a delay in the routine appointment given by our interventional radiologists based on the judgment of primary ENT surgeons given limited services available from the radiology department due to the COVID-19 uproar. Exclusion criteria for both groups include subjects who refused the procedures and patients who underwent prior fine-needle aspiration or core needle biopsy. The procedure details, advantages, and potential complications were thoroughly explained, and all subjects provided written informed consent. The Institutional Review Board approved this study of our institution (20201025-9163).

### Procedure

**Fig. 1:**
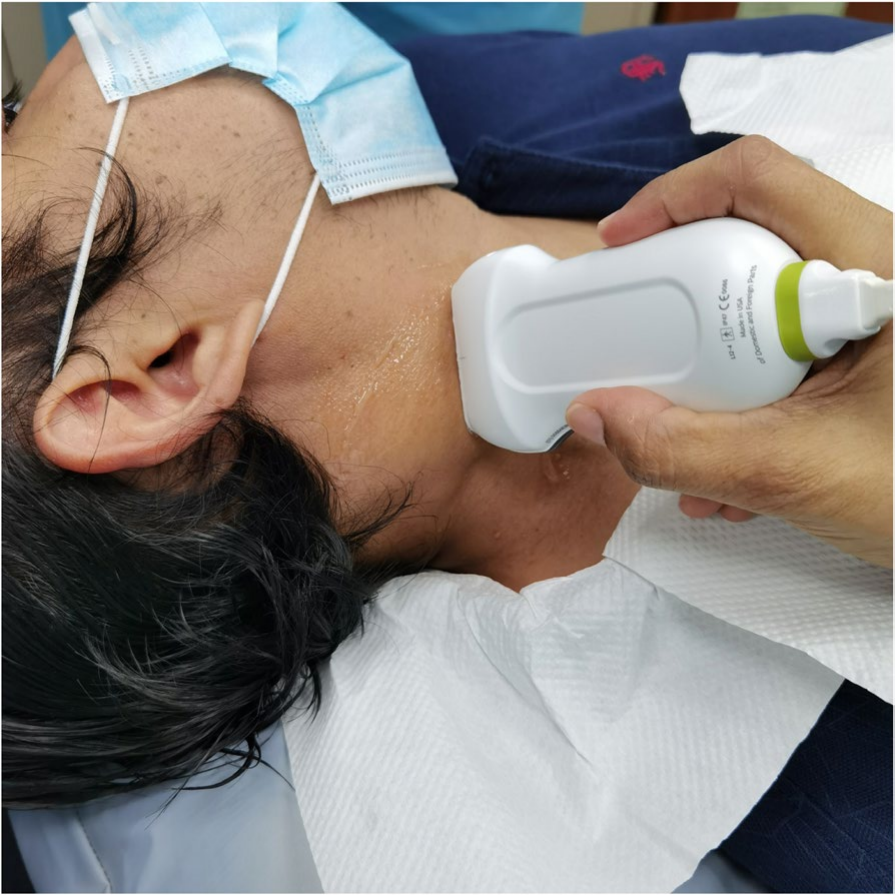
Surgeon performing USG using portable Philip system high-resolution ultrasound with broadband linear array

Prior to the ultrasound, a complete medical history and physical examination of the head and neck were performed on all subjects. Two ENT surgeons performed US and US-guided diagnostic or therapeutic procedures after thoroughly reviewing all relevant imaging studies. The portable Philip system high-resolution ultrasound with broadband linear array transducer 12 to 4 MHz was used in the study (Fig. 1). A thorough evaluation of the neck pathologies was performed, including the number, location, distribution, size, and ultrasonographic (US) features of the enlarged lymph nodes or masses. A more detailed US features of the target mass were performed by noting the size, area of solidity, vascularity, and safety of the needle passage route. A similar assessment was done for pre-therapeutic procedures to allow real-time and precise needle placement.

**Fig. 2:**
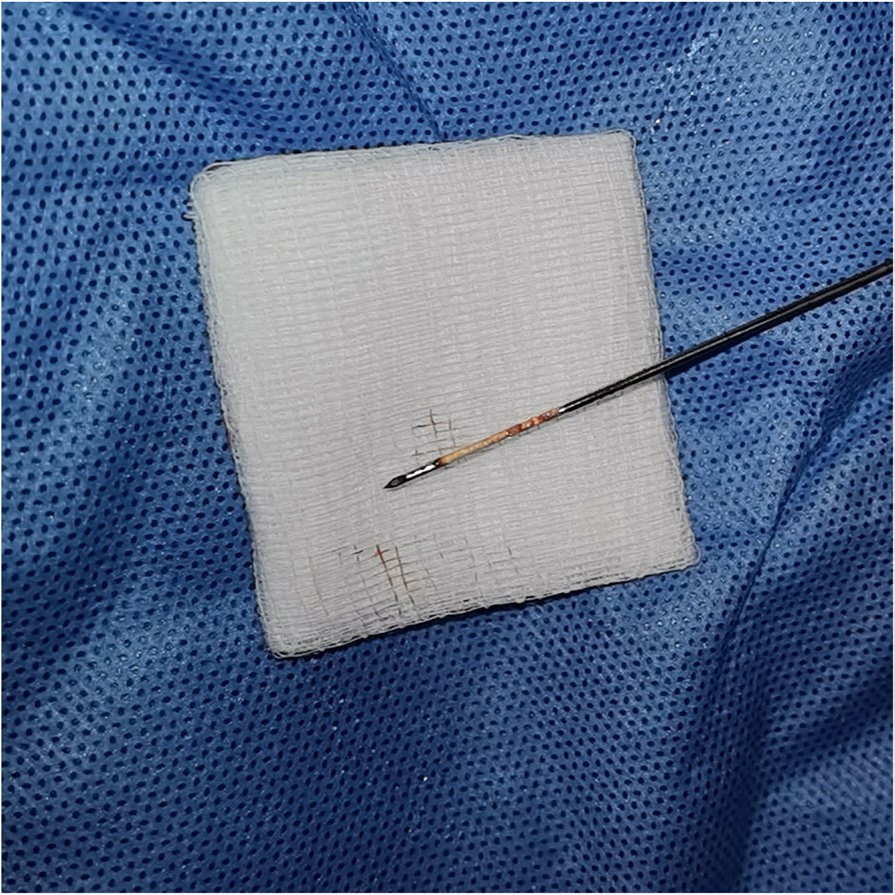
Examination of the gross core specimen by the operator

For core needle biopsy (CNB), an 18-gauge semi-automated biopsy needle (TSK Acecut; Create Medic, Yokohama, Japan) with a 1.1-cm excursion was used. The patient was placed in a supine position with the neck extended. When the targeted lymph nodes or masses in the lateral neck compartment were identified, the patient’s head was rotated to the contralateral side. One to four needle passes were made for CNB. The number of CNB specimens was determined by examining the gross core specimen by the operator (Fig. 2). After CNB, the patient was instructed to apply constant pressure to the puncture site for 30 min, and a repeated ultrasound was performed 30 min later to evaluate for post-procedural hemorrhage. The tissue cores were preserved in formalin for histopathologic examination and saline for immunohistochemical and molecular biological studies. Histopathological reports were issued by several pathologists working in our institution. The procedure was done in an outpatient clinic setting with strict physical distancing practice compliance, at least 1 m. Only a maximum of five personnel were allowed to be in the procedure room, including the surgeons and the assistants. Patients were obligated to use a surgical mask throughout the procedure. Furthermore, healthcare teams were required to use a surgical mask (N95), face shield, and proper hand hygiene during interaction with patients. A successful SUS is defined as the diagnostic procedure’s ability to facilitate the final histopathological diagnosis that can guide the subsequent treatment and disease resolution following the therapeutic procedure without the need for more invasive open surgical intervention.

## Results

We have identified 34 patients, but only 10 fit into our inclusion criteria. Out of the 10 subjects identified, 6 are males, and 4 are females, with a mean age of 49 years old. There were 5 diagnostic and 5 therapeutic SUS. The average duration of procedures recorded was 12 min. With our hospital’s strict COVID-19 prevention protocol, neither the operators (34 health staff) nor the 10 study subjects contracted the infection at least 30 days post-procedure. There was 0 complication recorded. At the first attempt, all subjects had successful tissue sampling or intervention for diagnostic and therapeutic SUS. The authors managed to perform both diagnostic and therapeutic procedures about 17.2 days earlier than the routine appointment from our hospital radiology counterpart. On the other hand, more urgent therapeutic procedures such as neck abscess aspiration were done 5 days earlier.

**Fig. 3:**
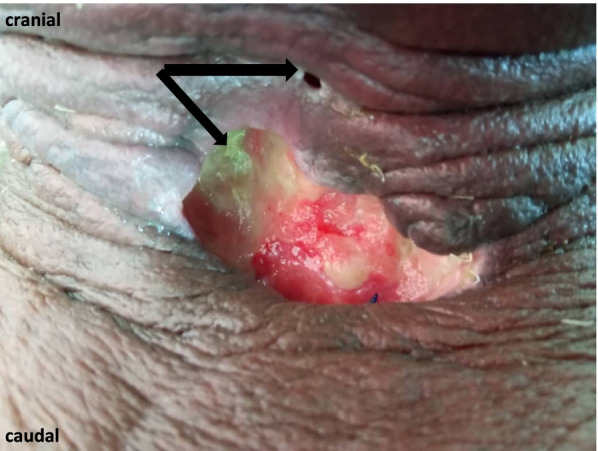
Pharyngocutaneous fistula (black arrow)

As for the outcome, our study reported an 80% success rate to initiate treatment or treatment cure rate. The basic demographic and clinical characteristics of the subjects are shown in Table 1. There was one failure in both groups. One subject required an open excision while the other had a failed therapeutic SUS, but it was expected due to multiple other contributing factors. He developed post-total laryngectomy pharyngocutaneous fistula (Fig. 3), and a bilateral submandibular gland Botox A injection was performed as part of its adjunct treatment. However, the ongoing active actinomycosis of the pharynx and concurrent neoadjuvant therapy for his concurrent rectal carcinoma likely impeded his healing process. A detailed clinical data of our study subjects are shown in Table 2.

**Table 1:** Patient characteristics and procedures

Male:female (mean age, years)	6:4 (49)
Patients with	
Diagnostic procedure	5
Therapeutic procedure	5
Diagnostic procedures	
Parapharyngeal mass	1
Cervical lymphadenopathy	4^a^
Therapeutic procedure	
Deep neck abscess aspiration	2
Botox injection salivary glands	2
Pigtail insertion for delayed chyle leak post neck dissection	1
Procedure-related data	
Mean duration (min)	12.0
Diagnostic	5.5
Therapeutic	6.5
Mean number of staffs involved	3.4
Means duration from routine appointment^b^ (days)	17.2
Diagnostic	12.2
Therapeutic	5
Complications	Nil

^a^Location of node involved supraclavicular, deep level II, and submental

^b^Indicates the number of days SUS been performed earlier than the routine appointment by radiology counterpart

**Table 2:** Clinical data in 10 subjects

Sex/age	Pathology	Site of mass	Number of masses	Size of lesion	Procedure	Related factors	Follow-up outcome
F/21	Neck abscess	Submental	–	4 cm × 3 cm	Aspiration	Healthy	Resolved after 2 weeks with oral cefuroxime without open drainage
M/56	Pharyngocutaneous fistula post total laryngectomy	Left stoma	–	2 cm × 1 cm	Botox injection bilateral submandibular gland	Laryngeal carcinoma recurrence post radiation	Resolved 1 week later with intravenous tazocin
M/81	Pharyngocutaneous fistula post total laryngectomy	Upper stoma	–	3 cm × 3 cm	Botox injection bilateral submandibular gland	• Laryngeal carcinoma recurrence post radiation • Active actinomycosis infection • Concurrent rectal adenocarcinoma ongoing chemotherapy	Fistula persists
F/66	Delayed chyle leak post open nodal excision for non-Hodgkin lymphoma	Left supraclavicular and level IV	–	4 cm × 5 cm	Aspiration and pigtail insertion	Healthy	Resolved after 5 days with subcutaneous octreotide and diet modification
M/40	Left parapharyngeal abscess	Right upper jugular		4 cm × 4 cm	Aspiration	Diabetes mellitus	Resolved 2 weeks later with intravenous augmentin without open drainage
M/66	Metastatic node with lung carcinoma	Right supraclavicular	1	2 cm × 3 cm	Core needle biopsy	Newly diagnosed right lung adenocarcinoma	Node disappeared after 2 cycles of palliative chemo at second month
F/36	Parapharyngeal pleomorphic adenoma	Right parapharyngeal and parotid space	1	4 cm × 3 cm	Core needle biopsy	Healthy	Post excision final histopathological result is consistent, asymptomatic at third month follow-up
M/32	Cervical sarcoidosis	Right lateral jugular chain and posterior traingle	6	3 cm × 4 cm (largest)	Core needle biopsy	Healthy	Needed follow-up open excision to conclude the diagnosis as the core needle showed granulomatous lesion
F/71	Recurrent diffuse large B cell lymphoma	Submental	1	3 cm × 2 cm	Core needle biopsy	• Severe COVID infection 1 month ago • Known lymphoma in remission	Node reduced in size after chemotherapy at 1 month follow-up
M/21	Tuberculous lymphadenitis	Right level II	2	2 cm × 2 cm	Core needle biopsy	Healthy	Node disappeared after intensive phase of anti-tuberculous drugs

## Discussion

As technology improves and physicians of various specialities become more comfortable with ultrasound, its use is becoming more common across specialities. Surgeons routinely perform ultrasound in multiple areas, including trauma, breast, thyroid, and vascular surgery [7]. Several surgical specialities in other countries incorporate SUS, and some have this as part of their training. Traditionally, most US-guided procedures were performed by radiologists due to specific sonographic knowledge and access to ultrasound equipment [7] With the increasing popularity of high-quality smaller portable units, more and more US-related procedures are performed by surgeons in the clinic setting [8]. In the surgery literature, much has been published about the accuracy of SUS in the head and neck, particularly of thyroid nodules and salivary masses [9, 10]. However, little has been published on the feasibility of SUS of the neck during the acute COVID-19 pandemic, particularly in countries with limited resources. Many institutions are faced with the challenge of upholding and maintaining the standard of care while maintaining the safety of both health care personnel and patients.

Nevertheless, many routine medical practices have been changed to reduce the mitigation of the deadly virus, including using appropriate personnel protective equipment (PPE), reducing the length of the procedure, and limiting the number of health personnel involved. The mean duration of the procedures is 12 min, with therapeutic being slightly longer than diagnostic, with a mean duration of 6–7 min for the former and 5–6 min for the latter. In addition, the mean number of staff involved was 3–4 staff. As the virus infects people through inhaled and direct droplet contact, all staff involved were instructed to follow strict procedural guidelines and a warning signage usage outside the procedure room to prevent overcrowding. The patients involved must have completed their vaccination dose, and they were encouraged to perform a self-rapid test kit (RTK) antigen test as a screening tool before the procedure. Our study has proven safe, with no reported COVID-19 infection contracted by the patients and staff involved up to a month following the procedure.

The appointment date for the procedure has increased significantly, especially at our center, as many surgical procedures were delayed, especially during the initial period of the pandemic. By shortening the appointment for the diagnostic procedure, an earlier diagnosis can be made, hence fastening the subsequent treatment plan, particularly if suspected malignancy. SUS has been frequently reported as an effective therapeutic tool in head and neck diseases, particularly deep neck abscesses. It is an excellent alternative to open surgical drainage with a faster recovery rate and shorter hospital stay [11]. The overall hospital expenses were also lower in comparison. Traditionally, surgical drainage and concurrent intravenous antibiotic administration have been the mainstay of treating inflammatory deep neck collection, but this conventional method has disadvantages. Commonly the surgical drainage has to be done under general anesthesia in which the airway needs to be secured fiber optically or with a tracheotomy when it is significantly compromised. Neck incisions may predispose patients to a risk of neurovascular injury and a cosmetically undesirable scar. In rare circumstances, tumor spillage with seeding may be the worse consequence in an infected neck space due to malignancy. Recent reports have shown SUS as a safe and effective tool in managing peritonsillar abscesses with good outcomes and shorter waiting times [12]. Despite the COVID-19 challenge, we successfully performed SUS interventional procedure much earlier with a good outcome.

In the surgeon’s hands, ultrasound guidance is a natural extension of the physical examination, enhancing one’s capacity to detect, diagnose, and ultimately treat masses in the head and neck. It has been a decade since SUS showed a higher diagnostic rate and efficiency than a standard palpation-guided technique for head and neck masses in a randomized control trial [9, 13]. On the other hand, the non-diagnostic rates from various surgeon performed series ranged from 3.6 to 13%, which is also favorably to a series of radiologist performed results with a range of 8 to 23% [14–16]. The benefit of SUS, particularly by head and neck surgeons, is typically due to the superior understanding and visualizing of anatomy in relation to preoperative examination and imaging. A high positive diagnostic yield of 80% was achieved in our study. The only failure was the subject diagnosed with sarcoidosis following an open surgical biopsy after his initial CNB result, which showed chronic granulomatous inflammation without other distinctive features of a specific disease. Hence, the open diagnostic biopsy was performed to rule out other causes of granulomatous disease, particularly the potential malignant lymphoma [17]. Notably, some prominent granulomatous lesions may mask the morphologic changes in lymphomas. Conventionally, fine needle aspiration cytology for a cervical mass is the minimally invasive method that allows a fast diagnosis with high accuracy [18]. It was suggested that using an on-site cytologist could reduce the non-diagnostic rate [19]. However, on-site cytology availability requires prior arrangements for the cytologists to attend biopsies. Hence, patients need to set up additional appointments for dedicated biopsy sessions after their initial assessment, negating the potential convenience afforded by office-based ultrasound.

The authors are aware of the limitation of the small cohort in this study, precluding the evaluation of diagnostic accuracy parameters, such as sensitivity and specificity. Secondly, the retrospective nature of the research could not eliminate the selection bias. However, this is thus far the pilot study with an excellent preliminary outcome from both diagnostic and therapeutic SUS from an otolaryngologist perspective during the COVID-19 pandemic. Hence, the study implies the reduced need for consultant radiologists and cytologists in our outpatient clinic, thus showing the more crucial role of a surgeon with broad radiological knowledge. A routine radiology-based head and neck procedure is unexpectedly impractical in our large outpatient clinic with a heavy daily workload since the pandemic has changed our routine clinical practice. With limited surgeon time, the procedure is sometimes delayed or canceled for more urgent cases. We, therefore, are establishing the use of core-needle biopsy systems in our workup algorithm for head and neck diseases. It is also worth noting that SUS is a valuable skill to be included in the training of otolaryngologists.

## Conclusion

Working within the resource constraint during the COVID-19 pandemic, a SUS CNB was the ideal choice to facilitate the management of head and neck masses in our center. We believe our study will be an eye-opener for surgeons to incorporate this practice as part of the routine to improve and fasten the management of head and neck diseases, especially in developing countries.

## Data Availability

The datasets generated during and/or analyzed during the current study are available.

## References

[1] Flatman S, Kwok MM, Magarey MJ (2020). Introduction of surgeon-performed ultrasound to a head and neck clinic: indications, diagnostic adequacy and a new clinic model?. ANZ J Surg.

[2] American Academy of Otolaryngology — Head and Neck Surgery. Practice management resources. https://www.entnet.org/content/position-statement-surgeon-performed-neck-ultrasound. Accessed Nov 2021.

[3] Ridder GJ, Technau-Ihling K, Boedeker CC (2005). Ultrasound-guided cutting needle biopsy in the diagnosis of head and neck masses. Laryngoscope.

[4] Sklair-Levy M, Amir G, Spectre G, Lebensart P, Applbaum Y, Agid R, Lieberman S, Ben-Yehuda D, Sherman Y, Libson E (2005). Image-guided cutting-edge-needle biopsy of peripheral lymph nodes and superficial masses for the diagnosis of lymphoma. J Comput Assist Tomogr.

[5] World Health Organization. WHO Director-General's opening remarks at the Mission briefing on COVID-19 - 12 March 2020. https://www.who.int/director-general speeches/ detail/who-director-general-s-opening-remarks-at-the-mission-briefing-on-covid-19-12-march-2020. Accessed Nov 2021.

[6] COVIDNOW in Malaysia. https://covidnow.moh.gov.my. Accessed Nov 2021.

[7] Ahn D, Kim H, Sohn JH, Choi JH, Na KJ (2015). Surgeon-performed ultrasound-guided fine-needle aspiration cytology of head and neck mass lesions: sampling adequacy and diagnostic accuracy. Ann Surg Oncol.

[8] Elliott M (2019). Neck lump clinics: improving time to treatment. ANZ J Surg.

[9] Law MT, Taylor M, Bennett IC (2011). Surgeon-performed ultrasound-guided needle biopsy of the thyroid: a safe and effective diagnostic procedure. World J Endocr Surg.

[10] Haidar YM, Moshtaghi O, Mahmoodi A, Helmy M, Goddard JA, Armstrong WB (2017). The utility of in-office ultrasound in the diagnosis of parotid lesions. Otolaryngol–Head Neck Surg.

[11] Dabirmoghaddam P, Mohseni A, Navvabi Z, Sharifi A, Bastaninezhad S, Safaei A (2017). Is ultrasonography-guided drainage a safe and effective alternative to incision and drainage for deep neck space abscesses?. J Laryngol Otol.

[12] Hallak B, Graber S, Bouayed S, Teiga PS (2020). Advantages of otorhinolaryngologist performed transcervical ultrasonography in the management of peritonsillar abscess. Am J Otolaryngol.

[13] Robitschek J, Straub M, Wirtz E, Klem C, Sniezek J (2010). Diagnostic efficacy of surgeon-performed ultrasound-guided fine needle aspiration: a randomized controlled trial. Otolaryngol Head Neck Surg.

[14] Charous SJ (2004). An overview of office-based ultrasonography: new versions of an old technology. Otolaryngology—head and neck. Surgery..

[15] Milas M, Stephen A, Berber E, Wagner K, Miskulin J, Siperstein A (2005). Ultrasonography for the endocrine surgeon: a valuable clinical tool that enhances diagnostic and therapeutic outcomes. Surgery..

[16] Bhatki AM, Brewer B, Robinson-Smith T, Nikiforov Y, Steward DL (2008). Adequacy of surgeon-performed ultrasound-guided thyroid fine-needle aspiration biopsy. Otolaryngol—Head Neck Surg..

[17] Du J, Zhang Y, Liu D, Zhu G, Zhang Q (2019). Hodgkin’s lymphoma with marked granulomatous reaction: a diagnostic pitfall. Int J Clin Exp Pathol.

[18] Tandon S, Shahab R, Benton JI, Ghosh SK, Sheard J, Jones TM (2008). Fine-needle aspiration cytology in a regional head and neck cancer center: comparison with a systematic review and meta-analysis. Head Neck J Sci Specialties Head Neck.

[19] Baloch ZW, Tam D, Langer J, Mandel S, LiVolsi VA, Gupta PK (2000). Ultrasound-guided fine-needle aspiration biopsy of the thyroid: role of on-site assessment and multiple cytologic preparations. Diagn Cytopathol.

